# Supplementary material for: Maternal–Neonatal Outcomes of Obstetric Deliveries Performed in Negative Pressure Isolation Rooms during the COVID-19 Omicron Variant Pandemic in Taiwan: A Retrospective Cohort Study of a Single Institution
Source: J Clin Med. 2022 Sep 16;11(18):5441. doi: 10.3390/jcm11185441 (PMC9502114; doi:10.3390/jcm11185441)
Supplement: Supplementary file 1 [file jcm-11-05441-s001.zip › jcm-1885227-supplementary.pdf]

Table S1: Detailed information on maternal medical diseases in the study population

Maternal medical diseases	Delivery room (n = 21)	Isolated negative pressure room (n = 2)
Type II diabetes mellitus	1	1
Type I diabetes mellitus	1	0
Hyperthyroidism	3	1
Hypothyroidism	3	0
Chronic hypertension	2	0
Aklyosis spondylitis	1	0
Asthma	1	0
Atiphospholipid syndrome	2	0
Masthenia gravis	1	0
Pophyria	1	0
Glucose-6-phosphate dehydrogenase deficiency	1	0
Systemic lupus erythematosus	2	0
IgA nephropathy	1	0
Chronic hepatitis B	2	0

Table S2. Maternal surgical outcomes of pairing each case with the same indication of cesarean section.

*Maternal Outcome Paired with Indications of Cesarean Deliveries—**Previous uterine surgery***			
	Delivery room, n = 19	Isolated negative pressure room, n = 9	*p*-value
Blood loss[†] (ml)	600 (300–1800)	400 (200–800)	0.042[※]
Postpartum hemorrhage[◊] (%)	3 (15.8%)	0 (0.00%)	0.530
Operative time[†] (min)	91 (49–126)	93 (64–127)	0.980
*Maternal Outcome Paired with Indications of Cesarean Deliveries—**Malpresentation***			
	Delivery room, n = 7	Isolated negative pressure room, n = 2	*p*-value
Blood loss[†] (ml)	400 (300–800)	450 (400–500)	0.648
Postpartum hemorrhage (%)	0	0	-
Operative time[†] (min)	94 (63–103)	82.5 (75–90)	0.462
*Maternal Outcome Paired with Indications of Cesarean Deliveries—**Cephalopelvic disproportion***			
	Delivery room, n = 1	Isolated negative pressure room, n = 1	*p*-value
Blood loss[†] (ml)	500	300	-
Postpartum hemorrhage (%)	0	0	-
Operative time[†] (min)	84	73	-
*Maternal Outcome Paired with Indications of Cesarean Deliveries—**Dysfunctional labor***			
	Delivery room, n = 16	Isolated negative pressure room, n = 3	*p*-value
Blood loss[†] (ml)	400 (300–900)	500 (400–1000)	0.302
Postpartum hemorrhage[◊] (%)	0	1 (33.3%)	0.176
Operative time[†] (min)	77 (55–118)	84 (76–92)	0.283

*Maternal Outcome Paired with Indications of Cesarean Deliveries—**Severe preeclampsia***

	Delivery room, n = 3	Isolated negative pressure room, n = 1	*p*-value
Blood loss† (ml)	400 (400–600)	200	-
Postpartum hemorrhage (%)	0	0	-
Operative time† (min)	81 (39–92)	103	-

*Maternal Outcome Paired with Indications of Cesarean Deliveries—**Non-reassuring fetal heart rate tracing***

	Delivery room, n = 5	isolated negative pressure room, n = 2	*p*-value
Blood loss† (ml)	650 (500–1200)	900 (700–1100)	0.481
Postpartum hemorrhage◊ (%)	2 (40.0%)	1 (50.0%)	1.000
Operative time† (min)	63 (61–90)	93.5 (86–101)	0.121

† Mann–Whitney U-test; ◊ Fisher's exact test; ※ two-sided p-value <0.05

Figure S1. Detailed clinical information on newborns with neonatal respiratory distress.

No.	Maternal COVID-19 PCR test status	Gestational age (weeks) at birth	Delivery methods	Downes' score	Free flow oxygen use	Further respiratory support	Final diagnosis
**1**	Positive	38 5/7	Cesarean section	3	yes	NC, flow 1L/min	TTN
**2**	Positive	36 6/7	Cesarean section	4	yes	NIMV,FiO2: 40%	Congenital pneumonia
**3**	Positive	40 0/7	Vaginal delivery	3	yes	No	TTN
**4**	Positive	37 5/7	Cesarean section	4	yes	NCPAP, FiO2:20%	TTN
**5**	Positive	34 3/7	Vaginal delivery	5	yes	NIMV, FiO2:20%	TTN
**6**	Positive	38 4/7	Vaginal delivery	2	yes	No	TTN
**7**	Positive	36 0/7	Vaginal delivery	2	yes	Np	TTN
**8**	Positive	35 6/7	Cesarean section	3	yes	NC, flow 1L/min	TTN
**9**	Positive	34 5/7	Vaginal delivery	2	yes	No	TTN
**10**	Positive	39 6/7	Cesarean section	3	yes	No	TTN
**11**	Positive	35 3/7	Cesarean section	4	yes	NIMV, FiO2:20%	TTN
**12**	Positive	39 4/7	Vaginal delivery	2	yes	No	TTN
**13**	Positive	39 6/7	Vaginal delivery	4	yes	NC, flow 1L/min	MAS
**14**	Positive	39 0/7	Vaginal delivery	4	yes	NIMV, FiO2:30%	TTN
**15**	Positive	38 2/7	Cesarean section	3	yes	NC, flow 1L/min	TTN
**16**	Positive	39 6/7	Vaginal delivery	4	yes	No	MAS
**17**	Positive	36 4/7	Vaginal delivery	3	yes	NC, flow 1L/min	TTN
**18**	Positive	38 5/7	Cesarean section	4	yes	NCPAP, FiO2:20%	MAS
**19**	Negative	36 1/7	Vaginal delivery	4	yes	NIMV,FiO2: 20%	TTN
**20**	Negative	35 1/7	Vaginal delivery	2	yes	No	Stage I RDS
**21**	Negative	38 1/7	Vaginal delivery	5	yes	IMV, FiO2:35%	TTN
**22**	Negative	34 3/7	Cesarean section	4	yes	NCPAP, FiO2:20%	TTN
**23**	Negative	35 3/7	Cesarean section	4	yes	NIMV, FiO2:20%	TTN
**24**	Negative	38 0/7	Cesarean section	4	yes	NCPAP, FiO2:20%	TTN
**25**	Negative	37 0/7	Cesarean section	4	yes	NCPAP, FiO2:20%	TTN
**26**	Negative	35 0/7	Cesarean section	4	yes	NIMV, FiO2:20%	TTN
**27**	Negative	36 3/7	Vaginal delivery	3	yes	NCPAP, FiO2:20%	TTN
**28**	Negative	39 6/7	Vaginal delivery	4	yes	NCPAP, FiO2:20%	TTN
**29**	Negative	38 2/7	Cesarean section	3	yes	NCPAP, FiO2:20%	TTN
**30**	Negative	36 6/7	Vaginal delivery	4	yes	NIMV, FiO2:25%	TTN
**31**	Negative	35	Vaginal delivery	4	yes	IMV, FiO2:40%	Pulmonary hypertension
**32**	Negative	37	Cesarean section	4	yes	NIMV, FiO2:25%	TTN
**33**	Negative	37 4/7	Cesarean section	3	yes	No	TTN
**34**	Negative	38	Cesarean section	4	yes	NCPAP, FiO2:20%	TTN
**35**	Negative	39 1/7	Vaginal delivery	4	yes	NCPAP, FiO2:20%	TTN
**36**	Negative	38 2/7	Vaginal delivery	3	yes	No	TTN

IMV, invasive mechanical ventilation; MAS, meconium aspiration syndrome; NC, nasal cannula; NCPAP, nasal continuous positive airway pressure; NIMV, non-invasive mechanical ventilation; RDS, respiratory distress syndrome; TTN, transient tachypnea of the newborn
